# Grandmaternal smoking, asthma and lung function in the offspring: the Lifelines cohort study

**DOI:** 10.1136/thoraxjnl-2020-215232

**Published:** 2021-02-04

**Authors:** Gillian M Mahon, Gerard H Koppelman, Judith M Vonk

**Affiliations:** 1 Department of Pediatric Pulmonology and Pediatric Allergology, GRIAC Research Institute, University Medical Center Groningen Beatrix Children's Hospital, Groningen, The Netherlands; 2 Department of Epidemiology, GRIAC Research Institute, University Medical Center Groningen, Groningen, The Netherlands

**Keywords:** tobacco and the lung, asthma, paediatric asthma

## Abstract

**Background/objective:**

Limited research exists regarding the association between grandmaternal smoking during pregnancy and the risk for asthma and altered lung function in grandchildren. This study aimed to investigate this association in a three-generation design.

**Methods:**

37 291 participants (25 747 adults and 11 544 children) were included from the Lifelines study, a prospective longitudinal three generation cohort study in The Netherlands. Spirometry was available in 69.5% and 61.1% of the included adults and children. Logistic and linear regression were used to analyse the association between grandmaternal smoking during pregnancy and (1) asthma, (2) early childhood asthma (ie, onset before 6 years) and (3) lung function level. Maternal and paternal grandmaternal smoking were studied separately and the analyses were stratified by adult/child and by gender. The analyses were adjusted for gender, current smoking, birth variables and socioeconomic status.

**Results:**

In the adult population, maternal grandmaternal smoking during pregnancy was associated with a higher risk for asthma (OR (95% CI): 1.38 (1.06 to 1.79)), early childhood asthma (1.49 (95% CI 1.06 to 2.11)) and a lower FEV1/FVC% predicted (B (95% CI): −1.04 (−1.91 to −0.16) in men. These findings were not observed in a separate analysis of children that participated in this study. There was also no significant association between paternal grandmaternal smoking and asthma/lung function.

**Conclusion:**

Maternal grandmaternal smoking during pregnancy is associated with higher asthma risk and lower lung function in male grandchildren and a reverse effect in male grandchildren of subsequent generations. Our study highlights the deep-rooted effects of tobacco smoking across generations.

**Key messages**

**What is the key question?**

- To investigate the effect of tobacco smoking during pregnancy on the risk for asthma and lung function in second generation offspring.

**What is the bottom line?**

- Grandmaternal smoking during pregnancy is associated with a higher asthma risk and lower lung function in male grandchildren.

**Why read on?**

- The adverse effects of tobacco smoking are deeply rooted across generations and this awareness in clinical practice contributes to promoting life-long respiratory health.

## Introduction

Asthma is a highly prevalent disease reported to affect more than 358 million people worldwide in 2015.^1^ In utero exposure to tobacco smoke is a long identified risk factor not only for asthma, but also for childhood respiratory symptoms and lower lung function in the first generation offspring.^2^ In the Netherlands, a peak in the prevalence of women smoking was observed in 1970 (42%), which steadily decreased in 1990 (32%) and further decreased in 2017 (14.1%).^3^ It is difficult to define the historical trends of women smoking specifically during pregnancy, however it was reported that 46% of Dutch women of childbearing age (20 to 34 years) smoked in 1958.^4^ More recently in 2010, one-third of Dutch women smokers (6.3% of women) reported to have smoked during pregnancy exposing more than 11 000 fetuses per year.^5^

In experimental animal studies, the effects of smoking on respiratory health are not only seen in the directly exposed fetus but also in the indirectly exposed gametes that form the second generation offspring. Exposing pregnant rats to subcutaneous nicotine was associated with lung function decrements and male specific increased airway hyperresponsiveness to methacholine in both first (F1) and second (F2) generation offspring.^6^ In a more recent study, adult mice were exposed pre-conception and post-conception to secondhand cigarette smoke. An altered lung integrity and a higher incidence of allergic asthma was found in both F1 and F2 progeny compared with comparable progeny of adult mice exposed to filtered air.^7^ These studies raise the question whether the smoking-induced epigenetic traits, that are associated with asthma risk and/or the level of lung function, exercise intergenerational effects via the germ line.

While many human studies show the effect of smoking during pregnancy on F1 offspring,^8^ few and somewhat conflicting studies have assessed the intergenerational inheritance patterns seen in the F2 generation in animal models. One of the first human studies observed a higher risk for childhood asthma, specifically early childhood asthma, when maternal grandmothers smoked while pregnant. This risk was independent of maternal smoking.^9^ In 2014, Miller *et al* were unable to replicate these findings for maternal grandmaternal smoking; however they found a higher risk for childhood asthma in granddaughters when investigating the effect of paternal grandmaternal smoking during pregnancy.^10^ This paternal specific association was found insignificant in another more recent study.^11^ Further to this, two large Scandinavian studies investigated the risk of asthma outcomes in grandchildren as a result of maternal grandmaternal smoking during pregnancy. One study found an associated higher risk for asthma symptoms and medication usage at ages 36 months and 7 years^12^ while a more recent study found an associated higher prevalence of childhood wheeze and use of asthma medications.^13^ Maternal smoking is also known to be associated with a lower lung function in F1 offspring.^14 15^ However, only one study in humans investigated the effect of grandmaternal smoking during pregnancy on lung function in the F2 generation and no significant associations were found.^10^ Thus, when assessing the intergenerational effects of smoke exposure, research should account for gender specific effects, timing of symptoms (ie, early onset asthma vs later onset asthma), as well as potential smoking effects on lung function in the grandchildren.

The aim of this study was to investigate the association between grandmaternal smoking during pregnancy and asthma, early childhood asthma and lung function levels (FEV_1_, FVC and FEV_1_/FVC ratio) in grandchildren using the three generation Lifelines cohort study. We separately analysed early childhood asthma since previous studies showed that this phenotype seems to be most affected by grandmaternal smoking during pregnancy. Stringent criteria were used to define asthma specifically, in order to avoid the inclusion of non-asthmatic childhood wheeze; moreover, we investigated a population comprised of grandchildren with an extensive age range (4 years old to 50 years old) over a long time period (pregnancies between 1967 to 2013) which incorporated different trends in smoking prevalence. The role of gender was scrutinised by separately analysing paternal and maternal grandmaternal smoking and outcomes in both grandsons and granddaughters.

## Materials and methodology

### Study population

The data used in these analyses were collected as part of the prospective population based Lifelines cohort study which investigates 167 548 subjects from three northern provinces of The Netherlands.^16^ The Lifelines study was founded to facilitate research on chronic disease and healthy ageing and follows participants aged between 6 months and 93 years who were enrolled between 2006 and 2013.^17^ Subjects aged between 25 and 50 years were initially invited to participate via their general practitioner. Subsequently, family members of the participants (parents, partners and children) were also asked to participate resulting in the inclusion of two-generation or three-generation families. At baseline examinations, participants completed a questionnaire and had several clinical measurements including lung function. All participants were followed up by means of a questionnaire every 1.5 years and extensive physical examination every 5 years.^17^ All participants signed a written informed consent.

**Figure 1:**
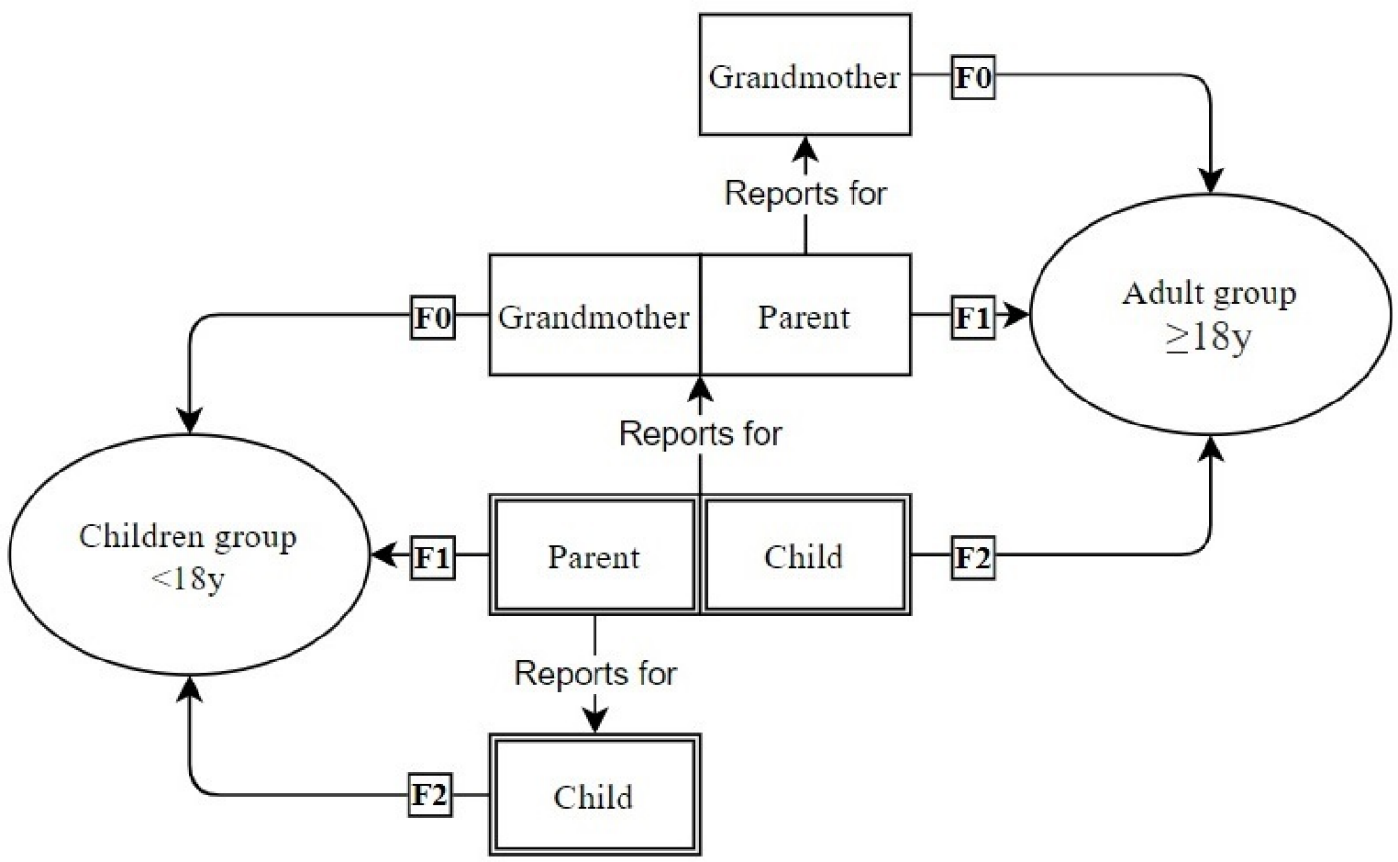
The different representation of three generations in both adult and children group and the roles of these generations in reporting information. F0, grandmaternal variables, for example, grandmaternal smoking; F1, maternal/paternal variables, for example, maternal smoking; F2, child variables, for example, asthma.

The questionnaires used in the Lifelines’ cohort differed for children (age <18 years) and adults. We therefore analysed children and adults separately. In both groups, grandchildren (F2) can be distinguished. For the F2 generation/grandchildren, we required the participation of at least one parent (F1) as these subjects report the smoking habits of the grandmothers during pregnancy (F0). Therefore, as figure 1 illustrates, the F1 generation in the children group can also be included as the F2 generation in the adult group. Unfortunately, detailed pedigree information and genetic profiles were not available and therefore we could not adjust for multiple (grand)children of the same (grand)mother.

In our analyses, children younger than 4 years of age (n=2411) were not included. This was due to the style of this first questionnaire where parents were asked to retrospectively report on asthma in their child before the age of 4 and as a result, data was either incomplete or missing because these children had not reached the age of 4. Also, adults older than 50 years (n=42 555) were excluded from our analysis since the parents of these subjects were not included in the cohort and therefore, data on grandmaternal smoking was not available. We conducted our analysis on the remaining population consisting of 122 582 participants.

### Outcome variables

Variables for the children and the adult group were defined separately. The questionnaires were completed by the parents in the children group and by the participants themselves in the adult group. These variables were defined within the constraints of the questionnaires. Asthma in the adult group was defined if one of the following two criteria was present : (1) Doctor diagnosed asthma ever or (2) At least two symptoms (wheeze, shortness of breath at rest, nocturnal shortness of breath based on the European Community Respiratory Health Survey-questionnaire (www.ecrhs.org)) and current use of at least one medication (ß-agonist, long acting ß-agonist/inhaled corticosteroid combination therapy, inhaled corticosteroid, anti-cholinergic, cromoglycate, theophylline, leukotriene receptor antagonist). If participants reported to have never been diagnosed with asthma nor having received treatment for asthma, they were included in the ‘no asthma’ group. Asthma in the children group was defined as ever having received treatment for asthma by a doctor. Participants who did not were included in the ‘no asthma’ group. We defined two asthma outcomes namely asthma and early childhood asthma. Asthma was the total group of those with asthma irrespective of age of onset. We defined early childhood asthma as having the first asthma attack before the age of 6 years. Pulmonary function was measured by trained Lifelines assistants with spirometry according to ATS (American Thoracic Society) guidelines using Welch Allyn V.1.6.0.489, PC-based SpiroPerfect with CardioPerfect Workstation software. A completed pulmonary function measurement consists of at least three technically correct manoeuvres. Spirometry was not performed in children younger than 8 years. Due to time constraints spirometry was performed in 70% of the participants. The subjects without lung function data were excluded from the analysis. The reference values of the Global Lung Function Initiative^18^ were used to calculate the % predicted values. We evaluated lung function based on the following measurements: FEV_1_/FVC ratio, FEV_1_ and FVC.

### Grandmaternal and maternal smoking variables

Grandmaternal smoking in both children and adults was defined if their mother and/or father reported that either the grandmother continued smoking as usual during pregnancy, smoked less or quit during (and not before) pregnancy. Smoking dose was not accounted for. In cases where smoking stopped before pregnancy, grandmaternal smoking was not assumed. Information on grandpaternal smoking was not included in the questionnaires. Maternal smoking in the adult group was defined if either of the following was reported: Mother continued smoking as usual during pregnancy, smoked less or quit during pregnancy. In the children group, maternal smoking was identified as present if the mother smoked during 1 to 3 or 3 to 6 or 6 to 9 months of the pregnancy. Absence of maternal smoking during pregnancy was specifically reported.

### Confounding variables

We adjusted our analyses for the following smoke exposure variables: maternal smoking during pregnancy, current smoking, former smoking and environmental smoke exposure in childhood. Birth variables were also adjusted for by including maternal age, birth weight, gestational age and history of breast feeding. We also adjusted for socioeconomic status (SES) and we defined this variable according to previous Lifelines associated research using income and education variables.^19^ Income was defined as net monthly household income and categorised into five earning brackets. Education was measured according to The Dutch Standard Classification of Education (SOI) and was categorised into four levels. For children we used the highest income and education of the parents. To avoid missing data in our covariates we recoded all missing values into an ‘unknown’ category.

### Statistical analysis

We conducted analysis using logistic regression where the primary outcomes were asthma and early childhood asthma and the predictor variable was grandmaternal smoking during pregnancy. Maternal grandmaternal smoking and paternal grandmaternal smoking were analysed separately. We used linear regression to analyse the association between grandmaternal smoking during pregnancy and lung function levels (expressed as % of predicted). Given the time trends of maternal smoking during pregnancy (low prevalence before 1950, followed by a gradual increase with the maximum prevalence around 1970) and in early childhood asthma/asthma (gradual increase in prevalence after the 1960s) we did not adjust for age since this may lead to over adjustment. We adjusted for gender, smoking exposure, birth variables and SES. The analysis was performed separately for children and adults due to different variable definitions. We also stratified the analyses by gender and by maternal smoking during pregnancy. A two-sided p value <0.05 was considered statistically significant. The analyses were performed using SPSS.^20^

## Results

**Figure 2:**
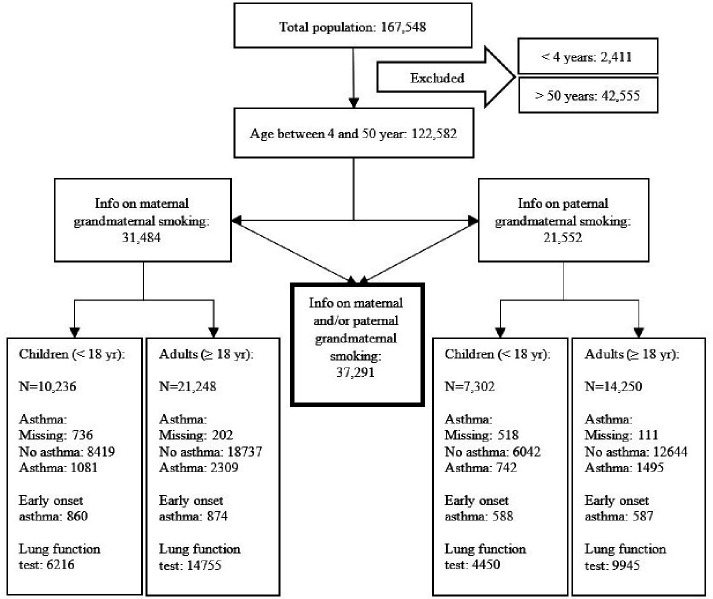
The selection of the population included in this study. First, participants were excluded based on age. Participants younger than 4 years of age or older than 50 years of age were excluded. Second, data spanning three generations was required to investigate a possible association between grandmaternal smoking during pregnancy and asthma in grandchildren. Therefore, participants who could not provide data on both childhood asthma and grandmaternal smoking were excluded. We investigated maternal grandmaternal smoking as well as paternal grandmaternal smoking and therefore those reporting on either one or both were included.

Of the 122 582 participants, we included 37 291 participants (11 544 children and 25 747 adults) who provided information on either maternal grandmaternal smoking and/or paternal grandmaternal smoking. (see figure 2) This reduction in data was due to the necessity of participation of multiple generations to determine grandmaternal smoking during pregnancy. The full comparison of the included and not included groups can be seen in the online supplement of this article (online supplemental table S1). Those included in the analyses were younger than those not included. They also had a higher prevalence of asthma particularly early childhood asthma and a lower prevalence of maternal smoking and childhood exposure to passive smoking compared with subjects not included in the analyses. Finally, the included subjects had a lower prevalence of current smoking, a higher mean birth weight and a higher prevalence of being breast fed than the not included subjects.

10.1136/thoraxjnl-2020-215232.supp1 Supplementary data

Table 1 outlines the characteristics of the included study population. The prevalence of asthma in both children and adult groups was comparable (11%) while a higher prevalence of early childhood asthma (<6 years) in the children group was observed (9.3% vs 4.4%). Maternal and paternal grandmaternal smoking during pregnancy was more prevalent in the children group while maternal smoking during pregnancy was more prevalent in the adult group.

**Table 1:** Descriptive statistics of subjects with information on gran-maternal smoking during pregnancy (maternal and/or paternal) stratified by age-group: children (>4 year and <18 year) and adults (≥18 year and ≤50 year)

	Children	Adults	P value
N	11 544	25 747	
Age, years	10.5 (3.7)	31.0 (8.4)	<0.001
Male gender	5638 (48.8)	9829 (38.2)	<0.001
Grandmaternal smoking			
Maternal	2501 (24.4)	1410 (6.6)	<0.001
Paternal	1374 (18.8)	686 (4.8)	<0.001
Maternal smoking	1226 (11.5)	4755 (20.8)	<0.001
Asthma	1214 (11.4)	2805 (11.0)	0.316
Early childhood asthma	967 (9.3)	1054 (4.4)	<0.001
Spirometry performed	7058 (61.1)	17 901 (69.5)	<0.001
FEV1 % predicted	95.2 (10.4)	95.2 (11.1)	0.732
FVC % predicted	96.3 (10.0)	98.6 (10.6)	<0.001
FEV1/FVC	0.86 (0.06)	0.80 (0.07)	<0.001
FEV1/FVC % predicted	98.2 (7.1)	96.0 (7.2)	<0.001
Active smoking			
Never	10 909 (95.7)	14 322 (58.7)	
Ex	98 (0.9)	4463 (18.3)	<0.001
Current	391 (3.4)	5616 (23.0)	
ETS in childhood			
No	7600 (71.0)	9831 (39.7)	
Yes	2045 (19.1)	14 785 (59.7)	<0.001
Unknown	1056 (9.9)	148 (0.6)	
Maternal age, years	29.8 (4.1)	26.9 (4.1)	<0.001
Birth weight, g	3499.1 (616.0)	3434.7 (580.9)	<0.001
Gestational age, years	39.6 (2.0)	39.8 (1.8)	<0.001
Breast feeding			
No	1967 (18.5)	6157 (25.0)	
Yes	8677 (81.4)	17 485 (71.0)	<0.001
Unknown	10 (0.1)	977 (4.0)	
Income			
<1000	167 (1.4)	4755 (18.5)	
1000–2000	1317 (11.4)	5297 (20.6)	
2000–3000	3763 (32.6)	6204 (24.1)	<0.001
>3000	5286 (45.8)	6708 (26.1)	
other	1004 (8.7)	2781 (10.8)	
Education			
Low	406 (3.5)	1613 (6.3)	
Intermediate	5579 (48.4)	14 359 (55.8)	<0.001
High	5499 (47.7)	9437 (36.7)	
Other/unknown	54 (0.5)	336 (1.3)	

ETS, environmental tobacco smoke.

In the adult group, maternal grandmaternal smoking was associated with a higher risk for both asthma and early childhood asthma (table 2). This higher risk was specifically observed in male grandchildren (asthma OR (95% CI): 1.38 (1.06 to 1.79) and early childhood asthma OR (95% CI): 1.49 (1.06 to 2.11)). When taking maternal smoking during pregnancy into account, a higher risk for early childhood asthma was present in the adult offspring of non-smoking mothers (OR (95% CI): 1.51 (1.12 to 2.05)), however, with adjustment this association was no longer significant probably due to lower sample size. There was no significant association between paternal grandmaternal smoking and any asthma outcomes (online supplemental table S2).

**Table 2:** Maternal grandmaternal smoking during pregnancy and risk for asthma and early childhood asthma in grandchildren. Stratified by gender and maternal smoking during pregnancy. Adjusted for gender, maternal smoking, current or former smoking, passive environmental smoke exposure in childhood, maternal age, birth weight, gestational age, breast feeding and socioeconomic status

	Children (<18 years)				Adults (≥18 years)			
	Asthma		Early childhood asthma		Asthma		Early childhood asthma	
	OR (95% CI)	P value	OR (95% CI)	P value	OR (95% CI)	P value	OR (95% CI)	P value
Total	1081 cases/8419 controls		860 cases/8419 controls		2309 cases/18 737 controls		874 cases/18 737 controls	
Unadjusted	0.92 (0.79 to 1.06)	0.233	0.95 (0.81 to 1.12)	0.569	1.29 (1.10 to 1.51)	0.002	1.55 (1.23 to 1.96)	<0.001
Adjusted	0.88 (0.76 to 1.03)	0.107	0.92 (0.78 to 1.09)	0.322	1.18 (1.00 to 1.39)	0.051	1.35 (1.06 to 1.71)	0.013
Females	478 cases/4381 controls		348 cases/4381 controls		1443 cases/11 623 controls		454 cases/11 623 controls	
Unadjusted	1.08 (0.87 to 1.34)	0.464	1.22 (0.95 to 1.55)	0.113	1.16 (0.94 to 1.42)	0.167	1.45 (1.05 to 2.01)	0.023
Adjusted	1.07 (0.86 to 1.34)	0.526	1.20 (0.94 to 1.54)	0.144	1.06 (0.86 to 1.31)	0.580	1.24 (0.89 to 1.73)	0.198
Males	603 cases/4038 controls		512 cases/4038 controls		866 cases/7114 controls		420 cases/7114 controls	
Unadjusted	0.79 (0.64 to 0.97)	0.026	0.79 (0.63 to 0.99)	0.043	1.54 (1.19 to 1.99)	0.001	1.73 (1.23 to 2.42)	0.001
Adjusted	0.74 (0.59 to 0.92)	0.006	0.74 (0.59 to 0.93)	0.011	1.38 (1.06 to 1.79)	0.016	1.49 (1.06 to 2.11)	0.023
Non-smoking mother	906 cases/7384 controls		742 cases/7384 controls		1608 cases/13 451 controls		616 cases/13 451 controls	
Unadjusted	0.88 (0.74 to 1.04)	0.143	0.96 (0.80 to 1.15)	0.657	1.21 (0.98 to 1.50)	0.077	1.51 (1.12 to 2.05)	0.007
Adjusted	0.86 (0.72 to 1.02)	0.088	0.93 (0.77 to 1.12)	0.439	1.14 (0.92 to 1.42)	0.228	1.32 (0.97 to 1.80)	0.076
Smoking mother	134 cases/950 controls		102 cases/950 controls		450 cases/3319 controls		160 cases/3319 controls	
Unadjusted	0.96 (0.66 to 1.39)	0.828	0.94 (0.62 to 1.43)	0.760	1.19 (0.88 to 1.61)	0.256	1.13 (0.69 to 1.85)	0.629
Adjusted	0.86 (0.58 to 1.26)	0.435	0.84 (0.54 to 1.31)	0.448	1.04 (0.77 to 1.42)	0.795	0.94 (0.57 to 1.56)	0.810

In the children group, a higher risk for asthma or early childhood asthma was not observed in the total group but instead a lower risk was seen in male grandchildren who were exposed to maternal grandmaternal smoking during pregnancy.

In both adults and children, there was no significant association between grandmaternal smoking and FEV_1_/FVC % predicted in the grandchildren. In the stratified analyses in the adult group, maternal grandmaternal smoking was associated with a lower FEV_1_/FVC % predicted in male grandchildren as well as in offspring of smoking mothers (table 3). In children, grandmaternal smoking was associated with a higher FEV1 % predicted and FVC % predicted in male grandchildren and with a higher FVC % predicted in grandchildren with non-smoking mothers (online supplemental table S3).

**Table 3:** Maternal grandmaternal smoking during pregnancy and FEV_1_/FVC % predicted. Stratified by gender and maternal smoking during pregnancy. Adjusted for gender, maternal smoking, current or former smoking, passive environmental smoke exposure in childhood, maternal age, birth weight, gestational age, breast feeding and socioeconomic status

	Children (<18 years)		Adults (≥18 years)	
	Beta-coefficient (95% CI)	P value	Beta-coefficient (95% CI)	P value
Total	n=6216		n=14 752	
Unadjusted	−0.32 (−0.75 to 0.1)	0.136	−0.3 (−0.79 to 0.2)	0.238
Adjusted	−0.15 (−0.57 to 0.28)	0.503	−0.19 (−0.68 to 0.30)	0.445
Females	n=3376		n=9163	
Unadjusted	−0.43 (−0.98 to 0.12)	0.123	0.20 (−0.39 to 0.79)	0.510
Adjusted	−0.23 (−0.78 to 0.32)	0.412	0.31 (−0.29 to 0.90)	0.311
Males	n=2840		n=5589	
Unadjusted	−0.18 (−0.84 to 0.49)	0.599	−1.15 (−2.02 to −0.28)	0.010
Adjusted	−0.06 (−0.73 to 0.60)	0.852	−1.04 (−1.91 to −0.16))	0.020
Non-smoking mother	n=5030		n=10 505	
Unadjusted	−0.29 (−0.78 to 0.20)	0.243	0.33 (−0.31 to 0.98)	0.308
Adjusted	−0.25 (−0.74 to 0.23)	0.308	0.25 (−0.39 to 0.89)	0.443
Smoking mother	n=710		n=2736	
Unadjusted	0.09 (−0.99 to 1.18)	0.867	−0.80 (−1.69 to 0.09)	0.077
Adjusted	0.03 (−1.07 to 1.13)	0.960	−0.91 (−1.81 to −0.01))	0.047

## Discussion

In this large population-based study in The Netherlands, the effects of maternal grandmaternal smoking during pregnancy on risk for childhood asthma differ between age-groups and therefore, time-periods. In the analysed adult group, maternal grandmaternal smoking during pregnancy was associated with a higher risk for both asthma and early childhood asthma in grandchildren. This risk for both asthma outcomes was consistently significant in grandsons while granddaughters only had a higher risk for early childhood asthma which attenuated with adjustment. Additionally, the risk for early childhood asthma was only seen in children of non-smoking mothers. In contrast, in the separately analysed children group, maternal grandmaternal smoking during pregnancy was associated with a lower risk for both asthma and early childhood asthma specifically in grandsons. Paternal grandmaternal smoking during pregnancy was not associated with any risk for asthma or early childhood asthma. For lung function, we found that maternal grandmaternal smoking during pregnancy was related to a lower FEV_1_/FVC % predicted in grandsons and in subjects with smoking mothers in the adult group. In the children group, grandmaternal smoking during pregnancy was associated with higher FEV_1_ % predicted and FVC % predicted in grandsons.

There are two main remarkable outcomes of this research. The first is the different outcomes observed between the two different groups, adults and children. The second is the profound male predominance, where asthma risk and lung function of male grandchildren are more significantly affected by grandmaternal smoking during pregnancy than that of female counterparts. Grandmaternal smoking during pregnancy seemed to exert a detrimental effect in those grandchildren of older generations (adult group), born approximately before the 1990s in this study, as these grandchildren had a higher risk for asthma and early childhood asthma. These results reinforce the positive association between grandmaternal smoking and childhood asthma under the age of 5 to 7 years described in three earlier studies.^9 12 13^ The study population included by Li *et al* is age comparable to the adult group in this study, although, the same cannot be said for the studied populations of Lodge *et al* and Magnus *et al*. However, the outcomes of these studies deviated from that of ours by focussing mainly on symptoms of asthma and/or use of asthma medication including sole usage of B agonist inhaler in childhood.

On the contrary, grandmaternal smoking during pregnancy exerted a protective effect in those grandchildren of younger generations (children group), born in the 1990s and onwards. This decreased risk for asthma and early childhood asthma, observed only in grandsons, is a new finding not previously reported in literature.

The contrasting results between the children and adult group in this study may be partly explained by the contrasting time periods associated with these groups, which allow for different trends in smoking habits and diagnosis of asthma. It can also be explained by the growing awareness of the adverse effects of smoking over the years and may have resulted in a healthy smoking family effect or in other words, lifestyle adaptations which contributed to an improved outcome for the generations that followed. This increasing awareness could also have caused selective reporting and recall bias of smoking during pregnancy.

Male grandchildren (F2) are more strongly affected by the effects of grandmaternal smoking compared with female grandchildren. The association between grandmaternal smoking during pregnancy and asthma in F2 males shown in this study’s adult population has been reported in studies of nicotine exposure during pregnancy on airway hyperresponsiveness in rats.^6^ Human studies have demonstrated this same predilection for males of the F1 progeny exposed to maternal smoking,^21 22^ However, until now this has not been demonstrated in F2 progeny. Gender and sex hormones are thought to affect lung development, and this can be seen clinically due to the male predominance of asthma prevalence early in childhood. The gender specific effect in response to grandmaternal smoking during pregnancy may be related to this male predisposition for unfavourable lung development.^23^ Conversely, the male specific protective effect of grandmaternal smoking during pregnancy seen in the children group of this study may relate to the study from Miller *et al*, where grandmaternal smoking, in the absence of maternal smoking, was associated with a more favourable birth weight in grandsons.^24^

The secondary findings in our study differ from results of previous research. Unlike Li and colleagues, we showed that early childhood asthma risk associated with grandmaternal smoking is only significant in those with a non-smoking mother. Grandmaternal smoking during pregnancy does not only expose the fetus (F1), but also the gametes that will form the next (F2) generation. Therefore, our results suggest that if the mother smokes during pregnancy, the adverse effects on the fetus may have reached a maximum effect and are not stronger in those with additional grandmaternal exposure. Further to this, this study compared early childhood asthma and asthma at any age and from this can conclude that the higher risk for asthma is not only confined to the early childhood asthma phenotype but also applies to risk for asthma in older years.

Paternal smoking habits and exposure prior to conception has been shown to increase the risk for asthma in offspring.^25^ Furthermore, paternal in utero smoke exposure (paternal grandmaternal smoking during pregnancy), has been linked with a higher risk for early childhood asthma specifically in granddaughters.^10^ However, in our study paternal grandmaternal smoking was not associated with asthma in grandchildren. Unfortunately, we were unable to investigate paternal smoking habits prior to conception and therefore, this interesting research question remains open for future study.

Grandmaternal smoking during pregnancy is associated with a lower lung function in grandchildren, specifically a lower FEV_1_/FVC % predicted. This is exclusively seen in male grandchildren and parallels the higher risk for asthma in this group. The ALSPAC study is the only study to our knowledge to have previously addressed this association and reported no significant associations between grandmaternal smoking and lung function.^10^

The strengths of this study are that asthma outcomes were defined as doctor diagnosed or doctor treated asthma. This may have reduced the inclusion of non-asthmatic wheeze or bronchitis in childhood. First, the questionnaires included numerous questions about a participants’ respiratory health. The asthma variable was constructed based on a response to a specific question about having asthma that was diagnosed by a doctor. Participants were asked separately about other respiratory conditions including wheezing, dyspnoea and bronchitis. The opportunity to report on various other respiratory illnesses alongside the specific reporting of asthma in these questionnaires should minimise misclassification. Second, we acknowledge that viral-induced wheeze may be confused for asthma in young children. In the attempt to exclude this group, the sole use of beta-agonist inhalers was not considered in defining asthma. While these approaches should minimise misclassification, we acknowledge that defining early onset asthma is difficult in an epidemiological setting.^26^ Furthermore, a comparative investigation of the outcomes of both early childhood asthma and asthma was performed, lung function was investigated as an outcome represented by objective measurements, and this study adjusted for many confounders including environmental smoke exposure which is omitted in many previous studies. The limitations of this study include the retrospective means of data collection on smoking during pregnancy and age of asthma onset which may be subject to recall bias or selective reporting, which may lead to an overestimation of the associations. This recall bias and selective reporting is difficult to avoid and would require long-running, prospective studies to eliminate this. However, while we can expect some bias when reporting respiratory symptoms in grandchildren and maternal smoking we expect that this bias is less evident when reporting respiratory symptoms in grandchildren and grandmaternal smoking, as this association is less likely recognised between these generations. Due to the limited availability of three generation families with complete data on grandmaternal smoking, we were unable to include the full cohort in our analyses. We acknowledge that including those only with information on grandmaternal smoking in our analyses may result in bias. The included population consisted of a greater number of younger (<18 years) and non-smoking participants and had a lower prevalence of maternal smoking during pregnancy and environmental smoke exposure during childhood and a higher prevalence of asthma and being breast fed compared with the non-included population. The significant differences in lung function, income and education between the included and non-included groups were very small. The difference in age between groups was inherent to the study design as we did not have information on grandmaternal smoking in subjects older than 50 years of age. In this study, a dose-response relationship could not be established since the amount of grandmaternal smoking during pregnancy was not available. Grandpaternal smoking prior to pregnancy was also not reported, therefore, we were unable to study the increased asthma risk in grandchildren associated with grandpaternal smoking before age 15 years.^11^ In addition, we could not investigate the effect of grandmaternal passive smoking during pregnancy as this data was not available. A final limitation is that we were unable to investigate third generation offspring (F3). The transgenerational effect of smoking has also been shown in F3 progeny animal studies.^27^ This mechanism of transgenerational inheritance is another potential important implication of smoking due to epigenetic effects that are inheritable across generations. Four-generational human studies are needed to investigate this which could be further strengthened by incorporating epigenetic investigations of biological matter itself.

## Conclusion

In this study, we have shown that maternal grandmaternal smoking during pregnancy exercises a detrimental role in pulmonary health in predominantly male grandchildren of less recent generations and conversely, a protective role in male grandchildren of more recent generations. This is represented by a higher asthma risk, paralleled by lower lung function and a lower asthma risk paralleled by favourable lung function, respectively. No association was found between paternal grandmaternal smoking during pregnancy and asthma in grandchildren. It is important to realise that the results of this observational cross-sectional study may be subject to bias which can only be eliminated by means of future prospective studies. Our study contributes to the growing awareness of adverse intergenerational effects of smoking^28^ and should stimulate further public health efforts to reduce smoking in the general population in particular before or during pregnancy.

## Data Availability

All data relevant to the study are included in the article or uploaded as supplementary information.
