# Supplementary material for: Grandmaternal smoking, asthma and lung function in the offspring: the Lifelines cohort study
Source: Thorax. 2021 Feb 4;76(5):441–7. doi: 10.1136/thoraxjnl-2020-215232 (PMC8070652; doi:10.1136/thoraxjnl-2020-215232)
Supplement: Supplementary data [file thoraxjnl-2020-215232supp001.pdf]

Supplementary Tables

	Information on grandparental smoking	Without information on grandparental smoking	P-value
N	37291	85291	
Age, years	24.7 (11.9)	40.2 (8.0)	<0.001
Male gender	15467 (41.5)	35239 (41.3)	0.600
Child, < 18 years	11544 (31.0)	931 (1.1)	<0.001
Maternal smoking	5981 (17.9)	14363 (21.6)	<0.001
Asthma	4019 (11.1)	8027 (9.6)	<0.001
Early onset asthma	2021 (5.9)	2188 (2.8)	<0.001
Spirometry performed	24959 (66.9)	61605 (72.2)	<0.001
FEV1 % predicted	95.2 (10.9)	95.3 (12.2)	0.037
FVC % predicted	98.0 (10.5)	99.6 (11.4)	<0.001
FEV1/FVC	0.82 (0.07)	0.78 (0.07)	<0.001
FEV1/FVC % predicted	96.6 (7.3)	95.4 (7.8)	<0.001
Active smoking			
Never	25231 (70.5)	39706 (49.1)	
Ex	4561 (12.7)	22194 (27.5)	<0.001
Current	6007 (16.8)	18940 (23.4)	
ETS in childhood			
No	17431 (49.1)	17872 (22.1)	
Yes	16830 (47.5)	62252 (77.2)	<0.001
Unknown	1204 (3.4)	563 (0.7)	
Maternal age, years	27.8 (4.3)	27.2 (4.7)	<0.001
Birthweight, gram	3455.4 (593.2)	3434.4 (642.5)	<0.001
Gestational age, years	39.7 (1.9)	39.7 (1.8)	0.586
Breastfeeding			
No	8124 (23.0)	20886 (26.0)	
Yes	26162 (74.2)	50588 (62.9)	<0.001
Unknown	987 (2.8)	8889 (11.1)	
Income			
<1000	4922 (13.2)	4394 (5.3)	
1000-2000	6614 (17.7)	16386 (19.6)	
2000-3000	9967 (26.7)	24737 (29.6)	<0.001
>3000	11994 (32.2)	25961 (31.0)	
other	3785 (10.2)	12157 (14.5)	
Education			
Low	2019 (5.4)	11582 (13.7)	
Intermediate	19938 (53.5)	46361 (54.6)	<0.001
High	14936 (40.1)	25590 (30.2)	
Other/Unknown	390 (1.0)	1300 (1.5)	

Table S1: Subjects with information on grandmaternal smoking during pregnancy (maternal and/or paternal) compared to subjects with no information on grandmaternal smoking.

	Children (< 18 years)				Adults (≥ 18 years)			
	Asthma		Early childhood asthma		Asthma		Early childhood asthma	
	OR (95% CI)	P	OR (95% CI)	P	OR (95% CI)	P	OR (95% CI)	P
Total	742 cases/6042 controls		588 cases/6042 controls		1495 cases/12644 controls		587 cases/12644 controls	
Unadjusted	1.08 (0.89;1.31)	.452	1.20 (0.97;1.48)	.087	1.21 (0.96;1.53)	.109	1.33 (0.94;1.88)	.107
Adjusted	1.05 (0.86;1.28)	.638	1.17 (0.95;1.44)	.152	1.14 (0.90;1.45)	.266	1.15 (0.81;1.64)	.436
Females	312 cases/3060 controls		219 cases/3060 controls		889 cases/7505 controls		278 cases/7505 controls	
Unadjusted	0.93 (0.69;1.26)	.645	1.16 (0.83;1.62)	.398	1.33 (0.99;1.78)	.058	1.56 (0.98;2.49)	.062
Adjusted	0.95 (0.70;1.29)	.736	1.14 (0.81;1.60)	.460	1.28 (0.95;1.72)	.108	1.36 (0.84;2.18)	.208
Males	430 cases/2982 controls		369 cases/2982 controls		606 cases/5139 controls		309 cases/5139 controls	
Unadjusted	1.19 (0.93;1.53)	.167	1.23 (0.94;1.60)	.126	1.04 (0.70;1.54)	.847	1.13 (0.67;1.90)	.646
Adjusted	1.13 (0.88;1.46)	.349	1.19 (0.91;1.56)	.203	0.97 (0.65;1.44)	.863	0.97 (0.57;1.65)	.914
Non-smoking mother	639 cases/5410 controls		516 cases/5410 controls		1051 cases/9104 controls		406 cases/9104 controls	
Unadjusted	1.02 (0.83;1.27)	.831	1.14 (0.91;1.43)	.262	1.29 (0.96;1.71)	.088	1.44 (0.94;2.19)	.096
Adjusted	0.99 (0.80;1.23)	.938	1.10 (0.87;1.39)	.417	1.25 (0.94;1.67)	.130	1.28 (0.83;1.97)	.264
Smoking mother	69 cases/572 controls		57 cases/572 controls		290 cases/2202 controls		118 cases/2202 controls	
Unadjusted	1.39 (0.80;2.40)	.239	1.59 (0.89;2.85)	.120	1.21 (0.76;1.93)	.426	1.36 (0.70;2.67)	.364
Adjusted	1.35 (0.76;2.41)	.305	1.57 (0.85;2.92)	.153	1.10 (0.68;1.77)	.693	1.20 (0.60;2.39)	.603

Table S2: Paternal grandmaternal smoking during pregnancy and risk for asthma and early childhood asthma in grandchildren. Stratified by gender and maternal smoking. Adjusted for gender, maternal smoking, current or former smoking, passive environmental smoke exposure in childhood, maternal age, birthweight, gestational age, breastfeeding and socioeconomic status.

	Children (< 18 years)				Adults (≥ 18 years)			
	FEV_1		FVC		FEV_1		FVC	
	B (95% CI)	P	B (95% CI)	P	B (95% CI)	P	B (95% CI)	P
Total	n=6216		n=6216		n=14752		n=14752	
Unadjusted	0.78 (0.17;1.39)	.013	1.07 (0.48;1.66)	<0.001	-0.49 (-1.24;0.26)	.200	-0.3 (-1.01;0.42)	.415
Adjusted	0.93 (0.31;1.55)	.003	1.05 (0.45;1.65)	.001	-0.05 (-0.80;0.71)	.901	0.09 (-0.63;0.81)	.809
Females	n=3376		n=3376		n=9163		n=9163	
Unadjusted	0.28 (-0.53;1.09)	.498	0.69(-0.11;1.5)	.092	-0.44 (-1.36;0.48)	.353	-0.74 (-1.63;0.15)	.103
Adjusted	0.26 (-0.56;1.08)	.541	0.45 (-0.36;1.26)	.277	0.15 (-0.78;1.08)	.751	-0.22 (-1.12;0.68)	.628
Males	n=2840		n=2840		n=5589		n=5589	
Unadjusted	1.40 (0.46;2.33)	.004	1.53 (0.66;2.41)	.001	-0.60 (-1.89;0.70)	.366	0.47 (-0.73;1.66)	.443
Adjusted	1.81 (0.86;2.75)	<0.001	1.85 (0.96;2.73)	<0.001	-0.31 (-1.62;0.99)	.637	0.7 (-0.51;1.9)	.260
Non-smoking	n=5030		n=5030		n=10505		n=10505	
Unadjusted	0.97 (0.27;1.68)	.007	1.22 (0.54;1.9)	<0.001	0.07 (-0.92;1.07)	.887	-0.38 (-1.32;0.57)	.434
Adjusted	0.97 (0.26;1.68)	.008	1.17 (0.49;1.85)	.001	0.37 (-0.63;1.37)	.466	0.06 (-0.88;1.01)	.895
Smoking mother	n=710		n=710		n=2736		n=2736	
Unadjusted	1.28 (-0.34;2.89)	.121	1.23 (-0.33;2.79)	.123	-1.20 (-2.52;0.11)	.073	-0.52 (-1.79;0.75)	.420
Adjusted	1.38 (-0.28;3.03)	.103	1.37 (-0.23;2.96)	.092	-0.74 (-2.07;0.59)	.276	0.10 (-1.18;1.39)	.873

Table S3: Maternal grandmaternal smoking during pregnancy and FEV_1 % predicted and FVC % predicted in grandchildren. Stratified by gender and maternal smoking. Adjusted for gender, maternal smoking, current or former smoking, passive environmental smoke exposure in childhood, maternal age, birthweight, gestational age, breastfeeding and socioeconomic status. B = Beta-coefficient.
